# Torin2 targets dysregulated pathways in anaplastic thyroid cancer and inhibits tumor growth and metastasis

**DOI:** 10.18632/oncotarget.3833

**Published:** 2015-04-14

**Authors:** Samira M. Sadowski, Myriem Boufraqech, Lisa Zhang, Amit Mehta, Payal Kapur, Yaqin Zhang, Zhuyin Li, Min Shen, Electron Kebebew

**Affiliations:** ^1^ Endocrine Oncology Branch, National Cancer Institute, National Institutes of Health, Bethesda, MD, USA; ^2^ Geisel School of Medicine at Dartmouth, Hanover, NH, USA; ^3^ Department of Pathology, University of Texas Southwestern Medical Center, Dallas, TX, USA; ^4^ Division of Discovery Innovation, National Center for Advancing Translational Sciences, National Institutes of Health, Bethesda, MD, USA

**Keywords:** torin2, mTORC1 inhibitor, mTOR, anaplastic thyroid cancer, quantitative high-troughput screening

## Abstract

Anaplastic thyroid cancer (ATC) is rare but it is one of the most lethal human malignancies with no effective therapy. There is a pressing need to identify new therapeutic agents for ATC. We performed quantitative high-throughput screening (qHTS) in ATC cell lines using a compound library of 3,282 drugs. qHTS identified 100 pan-active agents. Enrichment analysis of qHTS data showed drugs targeting mTOR were one of the most active drug categories, and Torin2 showed the highest efficacy. We found mTOR to be upregulated in ATC. Treatment of multiple ATC cell lines with Torin2 showed significant dose-dependent inhibition of cellular proliferation with caspase-dependent apoptosis and G1/S phase arrest. Torin2 inhibited cellular migration and inhibited the phosphorylation of key effectors of the mTOR-pathway (AKT, 4E-BP1 and 70S6K), as well as claspin and survivin expression, regulators of cell cycle and apoptosis. In our *in vivo* mouse model of metastatic ATC, Torin2 inhibited tumor growth and metastasis and significantly prolonged overall survival. Our findings suggest that Torin2 is a promising agent for ATC therapy and that it effectively targets upregulated pathways in human ATC.

## INTRODUCTION

Anaplastic thyroid cancer (ATC) is one of the most deadly solid human malignancies. It represents less than 2% of all thyroid cancers, has an annual incidence of 1-2 cases per million population per year but accounts for nearly one-third of thyroid cancer deaths [1, 2]. The median survival of patients with ATC is less than 6 month, and 90% of patients with ATC present with unresectable locally advanced or distant metastatic disease at time of diagnosis [3, 4]. There is currently no standard or effective therapy for ATC and the survival of patients with ATC has not improved in over 6 decades [3, 5]. Thus, there is an unmet need to identify effective agents for the treatment of ATC.

Drug discovery and development can be costly and take a long time to identify a clinically effective agent. For example, it has been estimated that to bring a single drug to market takes an average of 15 years and costs approximately $800 million [6]. Therefore, quantitative high-throughput screening (qHTS) of existing drugs has emerged as an attractive alternative, especially for orphan cancers such as ATC [7-9]. Screening a drug library has several advantages, which include knowledge of the drugs pharmacokinetic, pharmacodynamics and toxicity profile. This information could lead to a more efficient repurposing of drugs for rare cancers. Another important advantage of screening a Food and Drug Administration (FDA) approved drug library with known mechanism of action, is the ability to determine whether compound(s) found to be active on qHTS can target molecular pathways that are altered in human malignancies.

In this study, we performed qHTS in multiple ATC cell lines, and identified active compounds that were enriched for drugs targeting the mTOR pathway. We then demonstrate that the mTOR pathway is upregulated in ATC with validation of the potent anticancer effects of an irreversible potent mTOR inhibitor, Torin2, *in vitro* and *in vivo*. Furthermore, we show Torin2 mediates caspase-dependent apoptosis while reducing the anti-apoptosis protein survivin, which is upregulated in ATC. Lastly, Torin2 also induced G1/S phase arrest in ATC cells resulting in decreased claspin protein levels, a protein required for DNA replication.

## RESULTS

### Quantitative high-throughput screening identifies mTOR inhibitors are active in ATC cells

**Figure 1:**
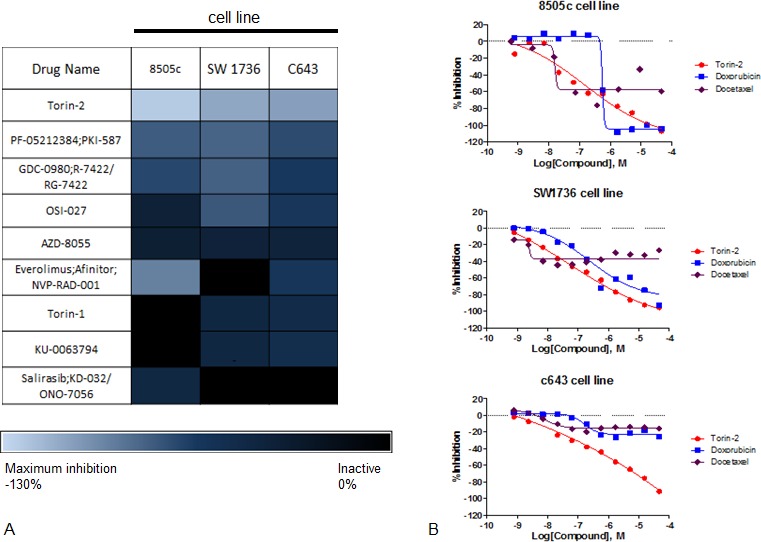
Results of qHTS in ATC cell lines **A.** Heatmap of efficacy analysis of drugs analyzed by qHTS in three ATC cell lines (8505c, SW1736 and C643), with maximum response (inhibition). **B.** Dose-response curves of Torin2, docetaxel and doxorubicin in ATC cell lines. The Y-axis shows percent inhibition and the X-axis shows the drug concentration.

We identified 100 active agents in the three ATC cell lines screened. Enrichment analysis by mode of drug action showed mTOR inhibitors to be one of the highest-ranking drug categories. Torin2 was the most active agent of nine mTOR inhibitors in all three ATC cell lines screened based on efficacy (maximum response >100%), best dose-response curve class, and the lowest half-maximum inhibitory concentration (IC_50_) (≤ 0.8μM) (Figure 1A). Although there is no effective chemotherapy agent for ATC, agents such as docetaxel and doxorubicin have been used as monotherapy or in combination [10-12]. Thus, we compared Torin2 activity in the three ATC cell lines to both agents and found Torin2 had better activity (maximum response and lower IC_50_) than docetaxel and doxorubicin (Figure 1B).

### Torin2 inhibits cellular proliferation in ATC cell lines, increases caspase activity and decreases cellular migration

**Figure 2:**
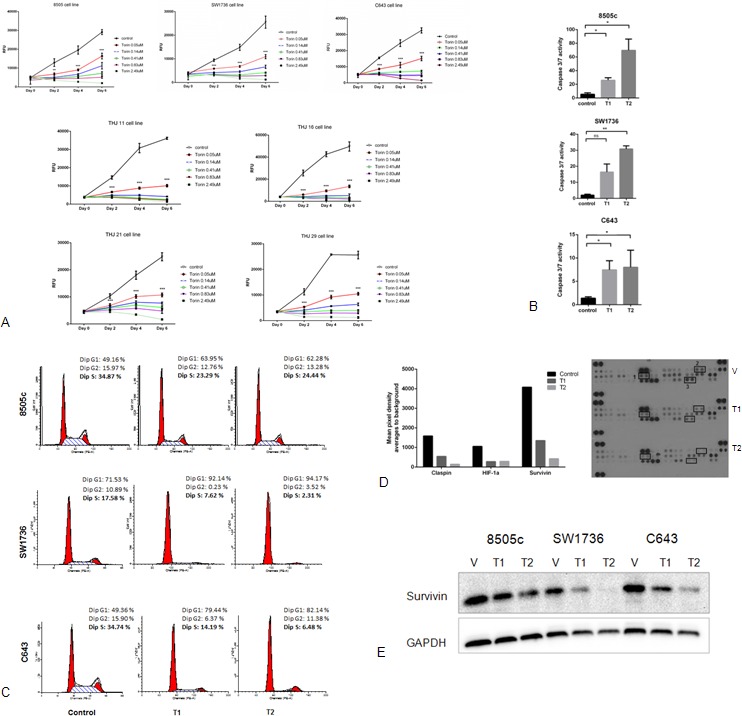
Effect of Torin2 on cellular proliferation, caspase activity and cell cycle in ATC cell lines **A.** Torin2 inhibits cellular proliferation in ATC cell lines. Cells were treated with Torin2 (0 to 2.49 μM) diluted in DMSO (control vehicle) for 6 days. RFU: Relative Fluorescence Unit. *p < 0.01, **p < 0.001, ***p < 0.0001. **B.** Torin2 increases caspase 3/7 activity in ATC cell lines. Caspase-Glo 3/7 assay was performed in three ATC cell lines after 48 hours of treatment using the two lowest concentrations of Torin2 used in the proliferation assays (Figure 2A, T1 = 0.05 μM and T2 = 0.14 μM).*p < 0.05, **p < 0.005, ns = non-significant. **C.** Cell cycle analysis was performed after 24 hours of treatment of Torin2. T1 = 0.05 μM and T2 = 0.14 μM. **D.** Effect of Torin2 on apoptosis-related proteins. ATC cells were treated for 48 hours using DMSO as control and Torin2 at T1 = 0.05 μM and T2 = 0.14 μM. A representative graph with corresponding scanned images is shown in Figure 2D for cell line C643. 1: HIF-1α, 2: Claspin, 3: Survivin. E. Western blot analysis of survivin after 48 hours of treatment with Torin2 at T1 = 0.05 μM and T2 = 0.14 μM.

*In vitro* studies using Torin2 in seven ATC cell lines showed a significant dose-dependent inhibition of cellular proliferation (Figure 2A). We found high concentrations of Torin2 were cytotoxic. Thus, we next asked whether Torin2 induced apoptosis. We found Torin2 increased caspase 3/7 activity, increased the number of cells in G1 and decreased the number of cells in S-phase (Figure 2B-2C), which is consistent with the effect on apoptosis [13].

To investigate the mechanism of how Torin2 induced apoptosis and G1/S-phase arrest, we analyzed the expression level of apoptosis-related proteins with an antibody array. We found that Torin2 reduced claspin, HIF-1α and survivin levels in a dose dependent fashion in all three cell lines, as shown in Figure 2D. Torin2 had a dose-dependent effect on survivin protein levels (Figure 2E).

**Figure 3:**
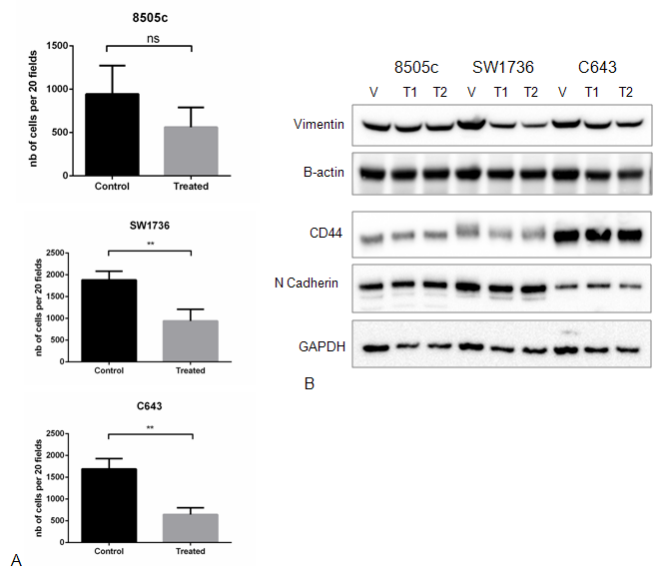
Effect of Torin2 on cellular migration and EMT marker expression **A.** Torin2 inhibits cellular migration. A transwell chamber assay was used to measure cellular migration with and without Torin2 treatment for 48 hours at 0.14 μM. *p < 0.05, **p < 0.005, ns = non-significant. nb on y-axis = number of cells. **B**. Western blots analysis of Epithelial-Mesenchymal-Transition (EMT) markers after 48 hours of treatment did not affect Vimentin, CD44 and N-cadherin protein levels. T1 = 0.05 μM and T2 = 0.14 μM.

We next investigated whether Torin2 had an effect on cellular migration as ATC is highly invasive and the mTOR pathway has been implicated in regulating cellular migration and epithelial-mesenchymal-transition (EMT), a feature omnipresent in ATC [14, 15]. Torin2 significantly inhibited cellular migration in 2 of 3 ATC cell lines, with a trend in 8505c cells when compared to control (Figure 3A). Given this effect on cellular migration, we evaluated whether Torin2 had an effect on proteins known to mediate EMT and found no significant effect on Vimentin, CD44 and N-cadherin protein levels (Figure 3B).

### Torin2 inhibits mTORC1 and the phosphorylation of mTOR-pathway related proteins

**Figure 4:**
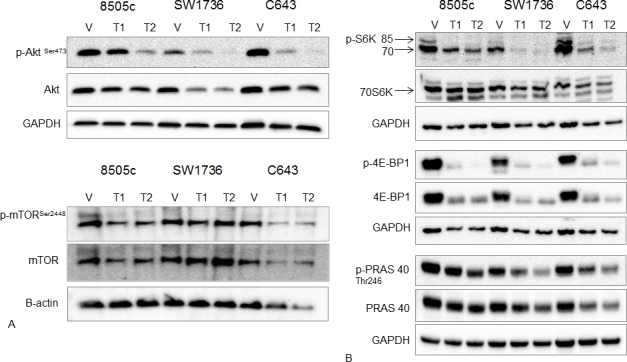
Effect of Torin2 on mTOR and mTOR-related protein expression and phosphorylation **A.** Western blot analysis of AKT, phospho-AKT^Ser473^, mTOR^Ser2448^ (mTORC1 site) and total mTOR. ATC cells were treated with Torin2 for 48 hours at T1 = 0.05 μM and T2 = 0.14 μM. Beta-actin was used as a loading control for the mTOR blot because of the higher molecular weight. **B.** Western blot analysis of downstream targets of mTOR: phospho-S6K (p-S6K), total 70S6K, phospho-4E-BP1 (p-4E-BP1), total 4E-BP1, phospho PRAS40 (p-PRAS40) and total PRAS40 with Torin2 treatment for 48 hours in ATC cells at T1 = 0.05 μM and T2 = 0.14 μM.

We next confirmed the inhibition of mTOR by Torin2 in ATC cell lines. Torin2 decreased phosphorylation of mTOR on Ser 2448, which is specific to the mTORC1 site and total mTOR levels (Figure 4A). Torin2 also decreased phosphorylation of AKT Ser473 and total AKT levels in a dose dependent fashion in all three ATC cell lines (Figure 4A). We next analyzed the downstream effectors of mTORC1, phospho-proteins 4E-BP1 and S6K [16, 17]. Torin2 showed a dose-dependent inhibition of phospho-4E-BP1 and S6K, and total 4E-BP1 in all 3 ATC cell lines; as well as a dose-dependent inhibition of phospho-PRAS40, which is a component and substrate of mTORC1 and a substrate of AKT (Figure 4B) [18].

### *In vivo* Torin2 inhibits tumor growth and metastases

**Figure 5:**
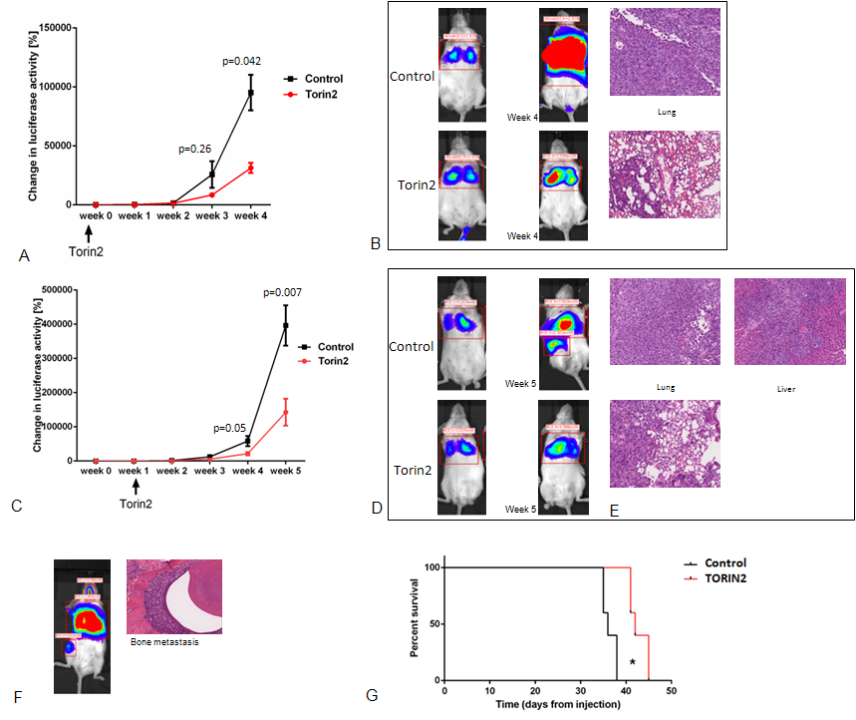
Effect of Torin2 on tumor growth and metastases *in vivo* **A.** and **B.** Pretreatment of mice with Torin2 decreases metastasis and tumor growth. Mice (*n* = 6) were treated by oral gavage 24 hours with either Torin2 20 mg/kg or Captisol before tumor engraftment of 8505c luc cells via the tail vein. **A.** Y axis in A indicates total change in luciferase activity per mouse as measured from the day of tumor cell injection and weekly. X axis shows the week after engraftment with the treatment timeline below. Results of lung tumor intensity, measured as change in luciferase activity in % per mouse (at 4 weeks *p* < 0.05, mean ± SEM). **B.** Representative whole mouse images of treated and control mice at 4 weeks after cell engraftment as well as representative H&E section of lung metastasis. **C.** and **D.** Torin2 inhibit growth and metastases *in vivo*. Mice (*n* = 12) were engrafted with 8505c luc cells via the tail vein and given daily oral gavage treatment with Torin2 20 mg/kg or Captisol at 1 week after the establishment of metastasis. **C.** Y axis indicates total change in luciferase activity in % per mouse as measured the day of tumor cell injection and then weekly (week 4, *p* = 0.05 and week 5, *p* = 0.007, mean ± SEM). **D.** Representative images of control and treated mouse with signal intensity for lung metastasis and at other sites of metastasis. **E.** Representative H&E images of Torin2 treated mice compared to the control mice (20X). A representative example of IVS image for signal intensity and the corresponding H&E for bone metastasis in a non-treated mouse is shown in **F. G.** Kaplan Meier survival curve with and without Torin2 treatment (*n* = 6 in each group). Median survival of 42 days in the Torin2 treated mice versus 36 days in the control mice (*p* = 0.0025, Mantel Cox log rank test).

Our *in vitro* data showed that Torin2 treatment inhibits ATC cellular proliferation, induces caspase-dependent apoptosis, decreased cellular migration, and effectively inhibits mTOR and mTORC1-related effectors of the mTOR pathway. Therefore, we investigated the effect of Torin2 treatment in a metastatic mouse model of ATC, which mimics the heavy tumor burden seen in human metastatic ATC [19]. We first evaluated if pretreatment of mice could reduce the rate of metastasis given the effects on cellular migration we observed *in vitro*. Torin2 significantly reduced ATC metastasis in mice pretreated with Torin2 (Figure 5A-5B). We then treated mice with established metastasis with Torin2. We found decreased growth and metastases with Torin2 treatment (Figure 5C-5F). The tumor burden was approximately one-third in the treated mice, with fewer mice developing diffuse metastases (liver and bone) with treatment (16% versus 67%) (Figure 5C-5F). Torin2 treatment in mice with established metastasis was also associated with longer survival time, median 1 week longer, (*p* < 0.005) (Figure 5G). We observed no toxicity with Torin2. Weekly weights were stable in both groups until week 4, and then decreased in the control group with increasing tumor burden.

### Torin2 targets are upregulated in ATC

**Figure 6:**
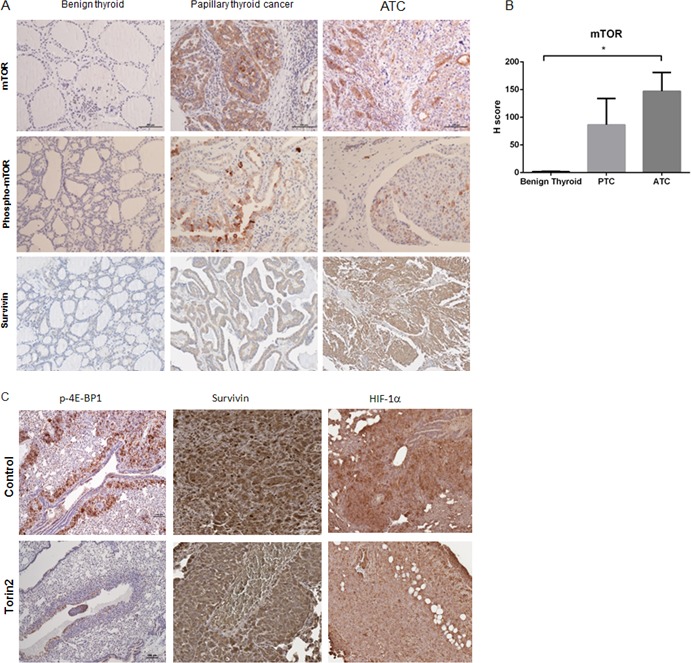
Torin2 targets are dysregulated in ATC **A.** Representative images of phospho-mTOR, mTOR and survivin staining in benign thyroid tissue, papillary thyroid cancer and ATC. **B.** Graph of H score of mTOR staining in 6 ATC tissues (1 was necrotic) (=12 sections), 6 PTC (=12 sections) versus 6 normal and 6 benign (follicular adenoma) (=24 sections) thyroid tissues, *p* = 0.013 (*), mean ± SEM. **C.** Representative images of whole mouse lung metastases at 4 weeks after cell engraftment with phospho-4E-BP1 staining. Representative images of survivin (reduced nuclear staining in the Torin2 group) and HIF-1α staining. Images at 20-100X.

Given that Torin2 had potent *in vitro* and *in vivo* effects in ATC, we next determined if the proteins targeted by this agent are dysregulated in ATC. Using human thyroid tissue arrays, we compared the staining of phospho-mTOR and mTOR in ATC versus normal and benign thyroid tissues. For mTOR, the H-score was significantly higher in ATC samples (the average readings of the duplicate sections showed an H-score of 1.6 in the normal and benign samples versus 147 in ATC samples, *p* < 0.05) (Figure 6A-6B). Additionally, we found Torin2 reduced survivin levels *in vitro* and therefore analyzed the expression of this protein in ATC. We found survivin protein expression to be positive in ATC and papillary thyroid cancer compared to normal thyroid tissue samples (Figure 6A), consistent with evidence that survivin overexpression is associated with progression to poorly differentiated thyroid cancer [20] and reduced survival in colorectal cancer [21]. These findings suggest that the targets of Torin2 are upregulated in ATC making it a rationale targeted agent for ATC. Further, we analyzed tumor samples from mice responding to Torin2 treatment and found reduced phospho-4E-BP1 staining, as well as reduced staining for survivin (reduced nuclear staining) and HIF-1α, a marker of aggressive cancer (Figure 6C) [22, 23].

## DISCUSSION

We performed qHTS in ATC cell lines and identified mTOR inhibitors to be one of the most active drug categories. Torin2 was the most active compound in ATC cells and effectively inhibited cellular proliferation, induced increased caspase activity and S-phase arrest, and inhibited cellular migration *in vitro*. In addition to inhibiting mTOR and the downstream effectors of the mTOR pathway (4E-BP1 and S6K), Torin2 inhibited the phosphorylation of AKT, an important signaling (PI3K/AKT/mTOR)-pathway in ATC and a variety of cancers [24]. *In vivo*, Torin2 inhibited growth and metastasis. Further, we show Torin2 to be more active than current therapies such as docetaxel and doxorubicin, and demonstrate that the targets of Torin2 are upregulated in ATC making it an attractive targeted therapy for ATC.

The mTOR-pathway controls cell survival and cell growth and is involved in multiple cancers and has been actively investigated as a target for cancer therapies [25, 26]. Several studies have investigated the mTOR-pathway in ATC. Papewalis and colleagues showed that everolimus inhibits the growth of ATC cell lines in a dose-dependent fashion [27] and Liu and associates have shown that phospho-mTOR is activated in ATC [28] consistent with our results. However, one of the shortcomings of using everolimus and other rapalogs to target the PI3K/AKT/mTOR-pathway is the development of resistance with long-term treatment as a result of reactivation of AKT and mTORC2 as these drugs target only mTORC1 [26, 29, 30]. More promising results have been observed in lung cancer and renal cell carcinoma models if the mTOR-pathway is targeted by inhibiting both AKT and mTOR [31, 32]. To overcome this limitation of AKT re-activation with everolimus and other rapalogs treatment, new second generation, ATP-competitive drugs have been developed that target both mTOR-complexes, mTORC1 and mTORC2, such as Torin2. Liu et al. [32] showed that Torin2 inhibits AKT and mTORC1, overcoming the feedback reactivation of AKT due to long term mTORC1 treatment, but it was effective in an *in vivo* lung tumor model only when combined with a MEK inhibitor. Simioni et al. [33] showed promising dual mTORC1 and mTORC2 inhibition with Torin2 in B-precursor acute lymphoblastic leukemia. Similar results have been reported in papillary thyroid cancer using a tumor flank xenograft model [34]. Lastly, a recent clinical report of a patient with metastatic ATC enrolled in a Phase II trial of everolimus therapy, showed resistance after an extraordinary 18 months of response. Whole-exome sequencing of pretreatment and drug resistant tumors revealed a mutation in mTOR that conferred resistance to allosteric mTOR inhibition (everolimus), but the mutation remained sensitive to a direct ATP-competitive mTOR kinase inhibitor such as Torin1 [35].

To our knowledge, this is the first study that shows Torin2 is active in ATC cells and in an *in vivo* mouse model of ATC that recapitulates the heavy tumor burden seen in patients with ATC [19]. We also show for the first time that Torin2 treatment in ATC cells results in lower claspin and survivin levels in ATC cells consistent with its effect on G1/S phase arrest and apoptosis, respectively [20, 36, 37]. Furthermore, Torin2 treatment *in vivo* was associated with reduced phospho-4E-BP1, HIF-1α and survivin protein expression in ATC responsive tumor samples. These data taken together show Torin2 to be potent in ATC suggesting it would be a good targeted therapeutic agent in patients with ATC.

In ATC, *TP53* inactivating mutations are most common, occurring in 42-55% of cases, followed by mutations in genes involved in the PI3K/AKT/mTOR, MAPK and WNT signaling pathways, mutations in *RAS* and *BRAF* (25%) [38, 39], and mutations in PIK3CA and PTEN, that occur in 17-23% and 12%, respectively, of ATC cases [38, 40]. Anti-BRAFV600E therapy has shown good activity in BRAF mutant cells *in vitro* and *in vivo* [41-43]. We found that Torin2 was effective in all cell lines harboring a similar mutation profile (*BRAF V600E*, *TP53*, *HRAS*, *PTEN*, *KRAS*, *RB*, *and PI3KCA*) as observed in human ATC tumor samples. This suggests that the activity of Torin2 is independent of the presence of driver mutations, which may activate the alternate MAPK pathway. We also identified claspin and survivin as new targets, which are dysregulated in ATC and that are reduced with Torin2 treatment in ATC cells. Survivin is upregulated in human ATC samples and is associated with more aggressive disease in differentiated thyroid cancer [20, 37]. These findings further support that Torin2 may be effective in patients with locally advanced and metastatic ATC.

In summary, we show that the mTOR-pathway is upregulated in ATC and that mTOR inhibitors are potent in ATC cell lines *in vitro* and *in vivo*. Torin2 effectively inhibits mTOR and mTOR-pathway effectors at multiple levels, and dysregulated protein targets in ATC such as survivin. Our findings taken together show that Torin2 is a good candidate drug for ATC treatment and should be considered for a clinical trial in patients with ATC.

## MATERIALS AND METHODS

### Quantitative high-throughput screening (qHTS) and compound library

The compound library consists of 3,282 small molecules that are either drugs that have been approved for human or animal use by the United States FDA or drug candidates that are currently in investigational use. Cell viability after drug treatment was measured using a luciferase-coupled ATP quantification assay (CellTiter-Glo®, Promega, Madison, WI) in three ATC cell lines: 8505c, C643, and SW1736. qHTS was performed as previously described [7, 9]. To determine compound activity in the qHTS assay, the titration-response data was plotted and modeled by a four parameter logistic fit yielding half maximum inhibitory concentration (IC_50_) and efficacy (maximal response) values. Raw plate reads for each titration point were normalized relative to a positive control defined as 100% inhibition (Tetraoctylammonium bromide, 100% inhibition) and DMSO only wells (defined as 0% inhibition). To assess the drug categories that were active in 8505c, C643, and SW1736 ATC cells, we performed enrichment analysis by the drug mode of action. The enrichment score was defined as the ratio of number of active drugs to the total of number tested drugs in that drug category (mode of action).

### mTOR inhibitor Torin2

Torin2 was purchased from Selleckchem (Houston, TX) for *in vitro* and *in vivo* experiments. The drug was diluted in DMSO for *in vitro* studies and in Captisol® (Ligand Pharmaceuticals, Inc., La Jolla, CA) for animal studies.

### Cell lines

Human ATC cell lines, 8505c (purchased from the European Collection of Cell Cultures, Salisbury UK), C643 and SW1736 (purchased from CLS Cell Lines Service GmbH, Germany), and THJ-11T, THJ-16T, THJ-21T and THJ-29T (kindly provided by Dr. John A. Copland III, Mayo Clinic, Jacksonville, FA) were maintained in Dulbecco's modified Eagle's medium (DMEM) supplemented with 10% fetal calf serum (FCS), penicillin (100 U/mL), streptomycin (100 μg/mL), fungizone (250 ng/mL), TSH (10 IU/L), and insulin (10 μg/mL) in 5% CO2 atmosphere at 37°C. All cell lines were authenticated by short tandem repeat profiling. The most relevant mutations profile of the ATC cell lines are 8505c (*BRAF V600E*, *TP53*), C643 (*HRAS*, *TP53*, *PTEN*), SW1736 (*BRAF V600E*, *TP53*, and *PIK3CB*), THJ-11T (*KRAS*), THJ-16T (*TP53*, *RB*, and *PI3KCA*), THJ-21T (*TP53*, *RB*, and *BRAF V600E*), and THJ-29T (*RB*) [44]. For 8505c, C643 and SW1736 targeted sequencing using the Ion Torrent TargetSeq platform (Life Technologies, Grand Island, NY) was done for known tumor suppressor and oncogenes.

### Cell proliferation, apoptosis and cell cycle assays

Cells were plated in 96-well plates and treated with Torin2 or vehicle control (DMSO) and the CyQUANT assay (Life Technologies, Grand Island, NY) was used to assess cell viability, according to the manufacturer's instructions on a SpectraMax M5 ® microplate reader (Molecular Devices, Sunnyvale, CA).

Caspase-Glo 3/7 assay (Promega, UK) was used to measure caspase activity after 48 hours of treatment according to the manufacturer's instruction.

For cell cycle analysis, cells were treated for 12, 24 and 48 hours. They were harvested, fixed with cold 70% ethanol for 30 minutes at 4°C, and then incubated in the dark with RNase (100 Mg/ml) and propidium iodide (50 Mg/ml) for 30 minutes at 37°C. A total of 20,000 cells were examined by flow cytometry using a Canto I flow cytometer (Becton-Dickinson, Franklin Lakes, NJ). Data were analyzed using ModFit software (Verity Software House, Topsham, ME).

### Migration assay

Cell migration was assessed using a Transwell chamber assay (BD Biosciences, San Jose, CA) according to the manufacturer's protocol. The lower chamber of the plate was filled with 750 μL DMEM supplemented with 10% FBS as a chemoattractant. After 22 hours of incubation at 37°C, the cells invading through the bottom surface of the inserts were fixed, stained with Diff-Quik (Dade Behring Newark, NJ), and photographed and counted using Image J software (NIH, Bethesda, MD).

### Western blot and antibodies

Total cell lysates were analyzed by SDS-PAGE, transferred to a nitrocellulose membrane, and immunostained with the following antibodies overnight at 4°C: anti-vimentin (1:1000, ab92547, AbCam, Cambridge, MA); anti-CD44 (1:1000, #5640, Cell Signaling Technology, Beverly, MA); anti-p-AKT Ser473 (1:1000, #9271, Cell Signaling Technology); anti-AKT (1:1000, #9272, Cell Signaling Technology); anti-p-mTOR Ser2448 (1:1000, #2971, Cell Signaling Technology); anti-mTOR (1:1000, #2972, Cell Signaling Technology); anti-p-p70S6K Thr421/Ser424 (1:1000, #9204, Cell Signaling Technology); anti-70S6K (1:1000, #2708, Cell Signaling Technology); anti-p-4E-BP1 Thr37/46 (1:1000, #2855, Cell Signaling Technology); anti-4E-BP1 (1:1000, #9452, Cell Signaling Technology); anti-p-PRAS40 (1:1000, #13175, Cell Signaling Technology), anti-PRAS40 (1:1000, #2691, Cell Signaling Technology), anti-Survivin (1:1000, #2808, Cell Signaling Technology), anti-N-cadherin (1:1000, #04-1126, EMD Millipore, Billerica, MA); and anti-human GAPDH (1:5000, sc-32233, Santa Cruz Biotechnology, Santa Cruz, CA) and anti-beta actin (1:5000, sc-81178, Santa Cruz Biotechnology) were used as loading controls. The membranes were incubated with the appropriate horseradish peroxidase-conjugated IgG (anti-rabbit 1:3000, Cell Signaling Technology, or anti-mouse 1:10000, Santa Cruz Biotechnology), and proteins were detected by enhanced chemiluminescence (ECL; Thermo scientific, Rockford, IL).

### Apoptosis array

The expression profile of apoptosis-related proteins was detected and analyzed using a human apoptosis array kit (ARY009), according to the manufacturer's instruction (R&D Systems, Minneapolis, MN). The membranes containing apoptosis-related antibodies were blocked with Array buffer for 1 h on a rocking platform and then incubated with lysates of untreated (DMSO) or Torin2 treated ATC cell lines (8505c, C643 and SW1736) overnight at 4°C. Chemiluminescent detection reagent was used after incubation with streptavidin-HRP conjugate. The membranes were scanned and pixel density was quantified by the mean of two replicate spot densities normalized to internal background using Image J software. Targets showing a two-fold difference in density to background ratio (increase or decrease) when compared to vehicle were selected.

### Immunohistochemistry

Tissue microarrays were purchased from US Biomax, Rockville, MD (#TH641) and included duplicate samples of 6 follicular adenomas, 6 follicular thyroid carcinomas, 6 papillary thyroid carcinomas, and 6 ATC, and 6 normal tissues from thyroid, 10 from lungs, testis, and adrenal pheochromocytoma. Slides were deparaffinized and rehydrated, and incubated with the primary antibody for phospho-mTOR Ser2448 and mTOR (Cell Signaling Technology) overnight as previously reported [45]. Staining intensity and percent of positive cells were analyzed and scored blinded by a clinical pathologist (P.K.). Further, mouse lung tissue slides from *in vivo* mouse studies were deparaffinized, rehydrated, and incubated with the primary antibody for phospho-4E-BP1 Thr37/46 (Cell Signaling Technology). The extent of staining (percentage of positive cell: 10/high power field) and intensity (0 for negative, 1 for weakly positive, 2 for moderately positive and 3 for strongly positive) were determined and an H score was assigned to each tissue section as a product of the extent of immunoexpression and the intensity of staining as previously described [45]. Additionally, anti-Survivin (NB500-201, Novusbio, Littleton, CO) at 1:500 dilution, and anti-HIF-1α ([ESEE122], ab8366, Abcam, Cambridge, MA) at 1:500 dilution were used for immunostaining using Vectastain ABC and DAB kits (Vector Laboratories, Inc. Burlingame, CA). Slides were scanned at cited magnification using a ScanScope XT digital slide scanner and viewed using ImageScope software (Aperio Technologies, Inc., Vista, CA).

### *In vivo* ATC metastasis mouse model

An *in vivo* ATC metastasis mouse model was used to assess the effect of Torin2 on growth and metastasis as previously described [19]. 8505c cells, stably transfected with a luciferase reporter gene *Luc2,* were injected into the tail vein of six- to eight-week-old NOD.Cg-*Prkdcscid Il2rgtm1Wjl*/SzJ mice purchased from The Jackson Laboratory (Maine, USA). The mice were maintained and bred according to the guidelines of the institute's Animal Advisory Committee. Immediately after tail vein injection, bioluminescence imaging was used to assess the injected cells in all mice using the Xenogen *in vivo* imaging system (Caliper Life Sciences Inc., Hopkinton, MA). Mice were injected intraperitoneally with 30 mg/mL of luciferin 15 minutes prior to imaging. Signal intensity was quantified as the sum of all detected photon counts within a region of interest using IVIS Living Image software (Caliper Life Sciences Inc., Hopkinton, MA).

To confirm tumor development in specific organ sites, ex vivo images were obtained. After whole body imaging, the mice were euthanized by CO2 inhalation and the organs were isolated and imaged again with the Xenogen system. Then the organs were fixed in formalin for histologic analysis. For animal survival analysis, mice were maintained under normal immunodeficient mouse care conditions, and monitored daily to assess their health status. Once the mice reached the humane euthanasia endpoints they were euthanized. Before euthanization, mice were imaged as described above.

### Statistical analyses

qHTS curve fitting and data analysis were performed as previously described [46]. Statistical analyses were performed using GraphPad Prism 5 software (GraphPad Software, La Jolla, CA). Parametric and nonparametric data were analyzed using a two-tailed t test and the Mann-Whitney U test, respectively. Survival analysis was performed using the Mantel Cox log rank test. *P* < 0.05 was considered statistically significant. Data are presented as mean ± standard deviation (SD) or mean ± standard error of mean (SEM).
